# Metabolic consequences of obesity on the hypercoagulable state of polycystic ovary syndrome

**DOI:** 10.1038/s41598-021-84586-y

**Published:** 2021-03-05

**Authors:** Abu Saleh Md Moin, Thozhukat Sathyapalan, Ilhame Diboun, Mohamed A. Elrayess, Alexandra E. Butler, Stephen L. Atkin

**Affiliations:** 1 Diabetes Research Center (DRC), Qatar Biomedical Research Institute (QBRI), Hamad Bin Khalifa University (HBKU), Qatar Foundation (QF), PO Box 34110, Doha, Qatar; 2 Academic Endocrinology, Diabetes and Metabolism, Hull York Medical School, Hull, UK; 3 Hamad Bin Khalifa University (HBKU), Doha, Qatar; 4 Biomedical Research Center (BRC), Qatar University, Doha, Qatar; 5 Royal College of Surgeons in Ireland Bahrain, Adliya, Kingdom of Bahrain

**Keywords:** Endocrinology, Endocrine system and metabolic diseases, Obesity, Pre-diabetes

## Abstract

Polycystic ovary syndrome (PCOS) women have a hypercoagulable state; however, whether this is intrinsically due to PCOS or, alternatively, a consequence of its metabolic complications is unclear. We determined plasma coagulation pathway protein levels in PCOS (n = 146) and control (n = 97) women recruited to a PCOS biobank. Circulating levels of a panel of 18 clotting pathway proteins were determined by Slow Off-rate Modified Aptamer-scan plasma protein measurement. Cohorts were age matched, though PCOS had elevated body mass index (*p* < 0.001), insulin (*p* < 0.001) and C-reactive protein (CRP) (*p* < 0.0001). Eight pro-coagulation proteins were elevated in PCOS: plasminogen activator inhibitor-1 (*p* < 0.0001), fibrinogen (*p* < 0.01), fibrinogen gamma chain (*p* < 0.0001), fibronectin (*p* < 0.01), von Willebrand factor (*p* < 0.05), D-dimer (*p* < 0.0001), P-selectin (*p* < 0.05), and plasma kallikrein (*p* < 0.001). However, two anticoagulant proteins, vitamin K-dependent protein-S (*p* < 0.0001) and heparin cofactor-II (*p* < 0.001) were elevated and prothrombin was decreased (*p* < 0.05). CRP, as a marker of inflammation, and insulin resistance (HOMA-IR) correlated with 11 and 6 of the clotting proteins, respectively (*p* < 0.05). When matched for BMI < 25 (16 PCOS, 53 controls) HOMA-IR remained elevated (*p* < 0.05) and heparin cofactor-II was increased (*p* < 0.05). In a multivariate analysis accounting for inflammation, insulin resistance and BMI, there was no correlation of PCOS with any of the coagulation proteins. The hypercoagulable state in PCOS is not intrinsic to the disease as it can be fully accounted for by BMI, inflammation and insulin resistance.

## Introduction

PCOS has been recognized as a reproductive-metabolic disorder given the excess prevalence of type 2 diabetes, hypertension, and cardiovascular diseases in this population at a later stage in life^1^. Polycystic ovary syndrome (PCOS) patients have increased platelet aggregation and decreased plasma fibrinolytic activity, resulting in a prothrombotic propensity^2,3^. Elevated coagulation markers have been reported in PCOS in comparison to controls^4^ and the coagulation parameters including prothrombin time, thrombin time and fibrin degradation products may be predictive of PCOS^5^. It has been reported that coagulation proteins such as thrombin-activatable fibrinolysis inhibitor, PAI-1, D-dimer, Antithrombin III and thrombomodulin are significantly increased in women with PCOS compared with age- and BMI-matched controls^4^. This suggests that PCOS, independent of its metabolic features, may be a risk factor for a hypercoagulable state.

This study was undertaken to determine the parameters contributing to the hypercoagulable state reported for PCOS.

## Materials and methods

We determined plasma coagulation pathway protein levels in PCOS (n = 146) and control (n = 97) women recruited to a PCOS biobank (ISRCTN70196169). The Newcastle & North Tyneside Ethics committee approved this study which was conducted according to the Declaration of Helsinki. All patients gave written informed consent. Clinical data and samples were accessed from the PCOS Genetic Biobank in the UK, therefore the Newcastle & North Tyneside Ethics committee serves as a national center to provide ethical approval for these Biobank samples.

All women were Caucasian. The diagnosis of PCOS was based on at least two out of three of the diagnostic criteria of the Rotterdam consensus as detailed previously^6^; namely clinical and biochemical evidence of hyperandrogenism (Ferriman-Gallwey score > 8; free androgen index > 4, total testosterone > 1.5 nmol/L), oligomenorrhea or amenorrhoea and polycystic ovaries on transvaginal ultrasound. Nonclassical 21-hydroxylase deficiency, hyperprolactinemia, Cushing’s disease and androgen secreting tumors were excluded by appropriate tests. The baseline study measurements have been described in detail previously^7^ and the demographic data for the PCOS and control women is shown in Table 1. All the control women had regular periods, no clinical or biochemical hyperandrogenism, no polycystic ovaries on ultrasound, no significant background medical history and none of them were on any medications including oral contraceptive pills or over the counter medications.

**Table 1:** Demographics, baseline, hormonal and metabolic parameters of the PCOS subjects and controls.

Baseline demographics	PCOS (n = 146)	Controls (n = 97)	*p* value
	Mean (SD)	Mean (SD)	
Age (years)	29.1 (6.1)	29.6 (6.5)	0.09
BMI (Kg/m^2^)	34.1 (7.5)	26.7 (6.6)	< 0.0001
Weight (Kg)	96.5 (23.7)	74.4 (18.4)	< 0.0001
Insulin (IU/ml)	10.2 (6.1)	6.2 (3.2)	0.001
HOMA-IR	3.8 (0.6)	1.6 (0.2)	< 0.005
CRP (mg/L)	4.4 (4.2)	2.4 (3.9)	0.0008
SHBG (nmol/L)	42.5 (39.6)	77.5 (78.4)	0.0003
Testosterone (nmol/l)	1.6 (1.0)	1.05 (0.48)	< 0.0001

*BMI* Body mass index, *HOMA-IR* Homeostasis model of assessment-insulin resistance, *CRP* C reactive protein, *SHBG* Sex hormone binding globulin.

Circulating levels of clotting pathway proteins were determined by Slow Off-rate Modified Aptamer (SOMA)-scan plasma protein measurement, the details of which have been previously reported^8^. Normalization of raw intensities, hybridization, median signal and calibration signal were performed based on the standard samples included on each plate, as previously described^9^.

We used version 3.1 of the SOMAscan Assay, specifically targeting those proteins involved in the coagulation cascade and fibrinolysis pathway in the SOMAscan panel of 18 proteins, in line with a previous report for those involved in a hypercoagulable state and alteration in coagulation proteins^10^: antithrombin III, heparin cofactor 2, fibrinogen gamma chain, D-Dimer, P-selectin, fibronectin, fibronectin fragment 3, fibronectin fragment 4, vitamin K dependent protein S, alpha 2 antiplasmin, fibrinogen, von Willebrand factor, plasma kallikrein, prothrombin, coagulation factor Xa, tissue factor, coagulation factor XI and angiostatin. Those proteins in the panel that inform us of a hypercoagulable state include fibrinogen and fibronectin as well as inhibition of the fibrinolytic pathway with plasminogen activator inhibitor-1 and evidence of activated fibrinolysis with D-Dimer levels^10–12^ (Table 2).

**Table 2:** Correlation of coagulation proteins with (A) Body mass index (BMI), (B) inflammation (C reactive protein; CRP) and (C) insulin resistance (HOMA-IR), (D) shows the results of the multivariate analysis taking into account BMI, CRP and HOMA-IR.

A. BMI			B. CRP			C. HOMA-IR			D. CRP + BMI + HOMA-IR		
	*p* value	r^2^		*p* value	r^2^		*p* value	r^2^		*p* value	r^2^
Antithrombin.III	< **0.00001**	0.154	Antithrombin III	< **0.00001**	0.202	Antithrombin III	< **0.00001**	0.140	D.dimer	0.06	0.151
Fibrinogen gamma chain	**0.00004**	0.101	Heparin cofactor.2	**0.00002**	0.116	Heparin cofactor 2	**0.0007**	0.070	Fibrinogen gamma chain	0.07	0.179
Vitamin.K dependent protein S	**0.0007**	0.070	Fibrinogen gamma chain	**0.00003**	0.111	P.selectin	**0.001**	0.066	Fibrinogen	0.07	0.089
D.dimer	**0.0008**	0.068	D.dimer	**0.00012**	0.094	Fibronectin	**0.009**	0.042	Plasma kallikrein	0.09	0.051
Fibronectin	**0.0008**	0.068	P.selectin	**0.0012**	0.067	Vitamin.K dependent protein.S	**0.015**	0.037	Fibronectin Fragment.4	0.12	0.064
Prothrombin	**0.007**	0.044	Fibronectin	**0.0014**	0.066	Alpha.2 antiplasmin	**0.02**	0.032	Vitamin.K dependent protein.S	0.14	0.089
Heparin cofactor.2	**0.009**	0.042	Fibronectin Fragment.3	**0.006**	0.050	Prothrombin	0.05	0.024	von Willebrand factor	0.14	0.035
Fibrinogen	**0.013**	0.038	Fibronectin Fragment.4	**0.007**	0.047	Fibronectin Fragment.3	0.09	0.018	Coagulation factor.Xa	0.19	0.030
Angiostatin	**0.014**	0.037	Vitamin K dependent protein.S	**0.02**	0.037	Plasma kallikrein	0.18	0.011	Antithrombin.III	0.19	0.264
Fibronectin Fragment.3	**0.03**	0.031	Alpha.2 antiplasmin	**0.02**	0.036	Tissue Factor	0.20	0.010	Fibronectin	0.24	0.094
P.selectin	**0.04**	0.026	Fibrinogen	**0.02**	0.035	Fibrinogen gamma chain	0.24	0.009	Fibronectin Fragment.3	0.31	0.060
Alpha.2 antiplasmin	0.16	0.012	von Willebrand factor	0.12	0.016	Fibronectin Fragment.4	0.32	0.006	Tissue Factor	0.40	0.030
Fibronectin Fragment.4	0.18	0.011	Plasma kallikrein	0.18	0.012	D.dimer	0.43	0.004	Angiostatin	0.41	0.056
Tissue.Factor	0.19	0.011	Prothrombin	0.19	0.011	Angiostatin	0.50	0.003	Prothrombin	0.42	0.048
von.Willebrand factor	0.26	0.008	Coagulation factor.Xa	0.22	0.010	Coagulation Factor.XI	0.81	0.000	Alpha.2 antiplasmin	0.42	0.052
Coagulation factor Xa	0.49	0.003	Tissue.Factor	0.71	0.001	Coagulation factor.Xa	0.83	0.000	Heparin cofactor 2	0.58	0.133
Coagulation Factor.XI	0.66	0.001	Coagulation Factor.XI	0.73	0.001	von Willebrand factor	0.87	0.000	Coagulation Factor.XI	0.70	0.005
Plasma kallikrein	0.97	0.000	Angiostatin	0.74	0.001	Fibrinogen	0.93	0.000	P.selectin	0.71	0.096

Bolded numbers indicate significant differences between PCOS and control groups, at the *p* < 0.05 level.

### Statistics

Measured protein data were log transformed to ascertain normality. Proteins were regressed on the continuous variables CRP, HOMA-IR and BMI in separate models to assess the extent of association with each trait. A multivariate linear model incorporating all three traits and PCOS status was performed to evaluate the relationship between the measured proteins and PCOS whilst correcting for the traits. All analyses were performed using R version 4. P values were corrected for multiple testing using the false discovery rate (FDR).

### Ethics approval

The Newcastle & North Tyneside Ethics committee approved this study which was conducted according to the Declaration of Helsinki. All patients gave written informed consent. Clinical data and samples were accessed from the PCOS Genetic Biobank in the UK, therefore the Newcastle & North Tyneside Ethics committee serves as a national center to provide ethical approval for these Biobank samples.

### Consent for publication

All authors gave their consent for publication.

## Results

Cohorts were age matched, though PCOS had elevated BMI (*p* < 0.001), fasting glucose (*p* < 0.05), insulin (*p* < 0.001), C-reactive protein (*p* < 0.0001) and platelet number (*p* < 0.01).

**Figure 1:**
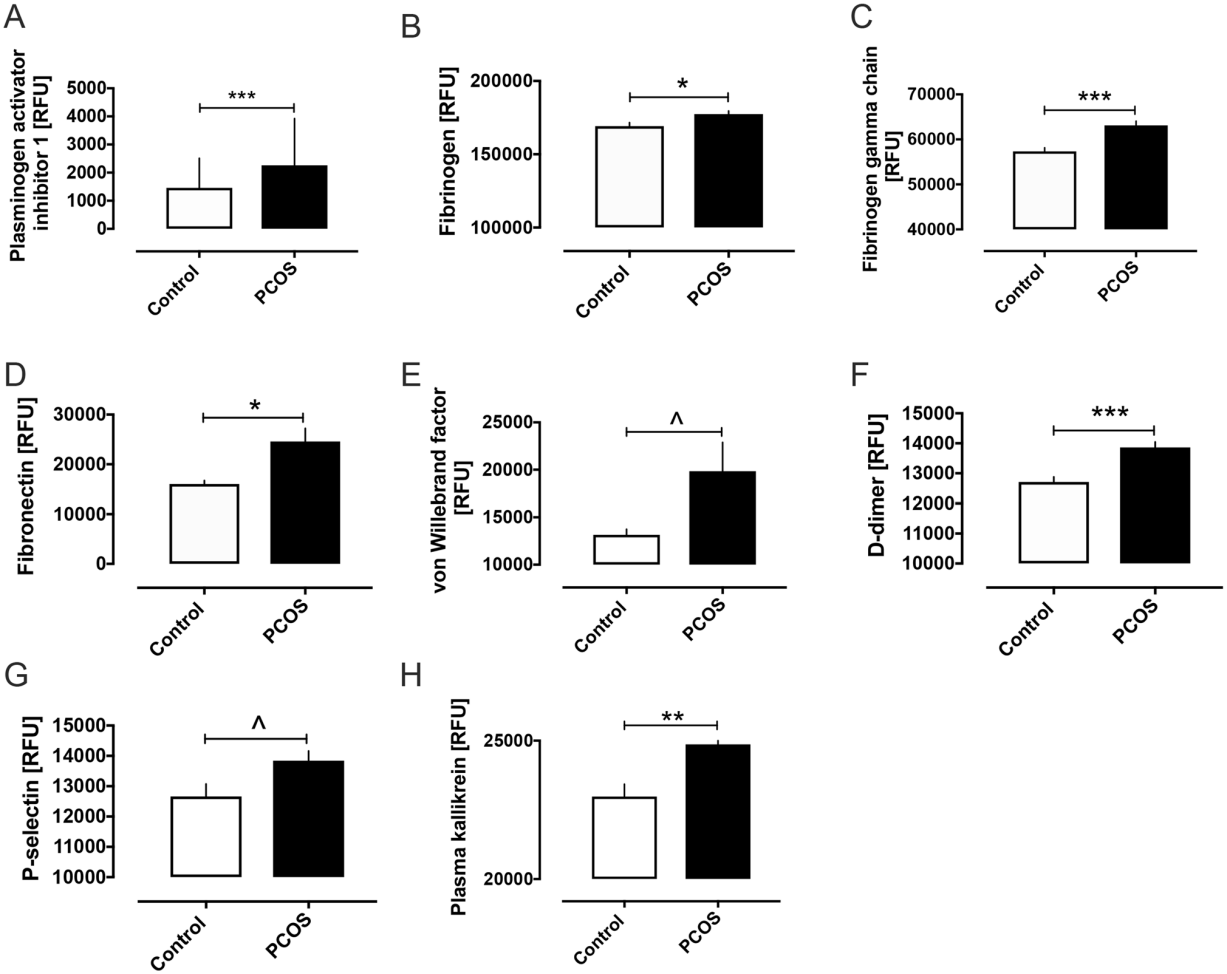
Pro-coagulation clotting pathway proteins that were increased in women with polycystic ovary syndrome (PCOS). Levels of plasma plasminogen activator inhibitor 1 (**A**), fibrinogen (**B**), fibrinogen gamma chain (**C**), fibronectin (**D**), von Willebrand factor (**E**), D-dimer (**F**), P-selectin (**G**) and plasma kallikrein (**H**) in women with and without polycystic ovary syndrome (PCOS). RFU, relative fluorescent units. Graphs shown as mean ± SEM. ^*p* < 0.05, *p < 0.01, **p < 0.001, ***p < 0.0001.

**Figure 2:**
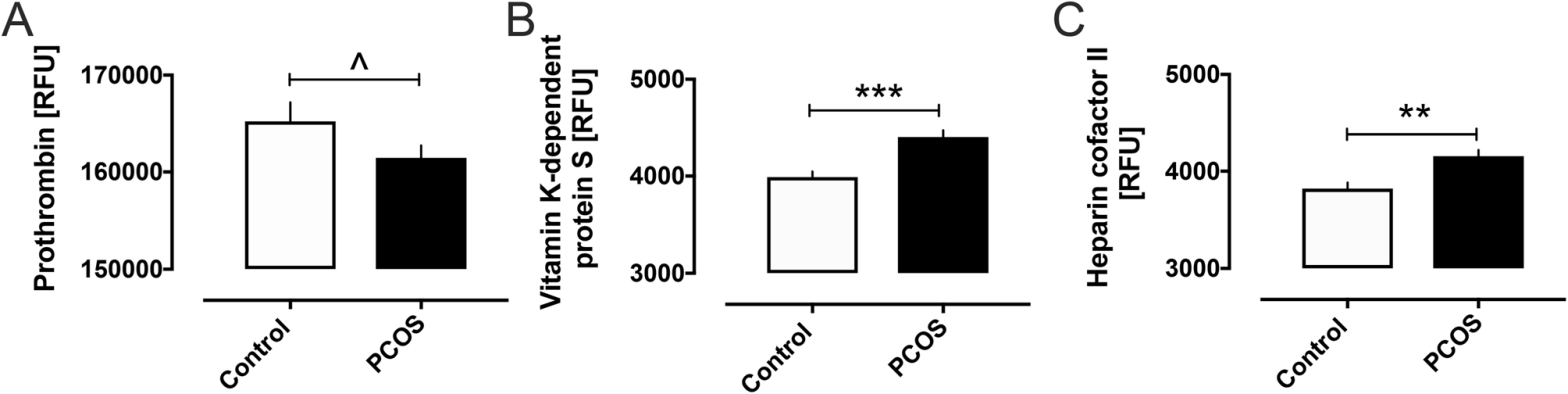
Anti-coagulation clotting pathway proteins that were altered in women with polycystic ovary syndrome (PCOS). Levels of plasma prothrombin (**A**), Vitamin K dependent Protein S (**B**) and heparin cofactor II in women with and without polycystic ovary syndrome (PCOS). RFU, relative fluorescent units. Graphs shown as mean ± SEM. ^*p* < 0.05, **p < 0.001, ***p < 0.0001.

**Figure 3:**
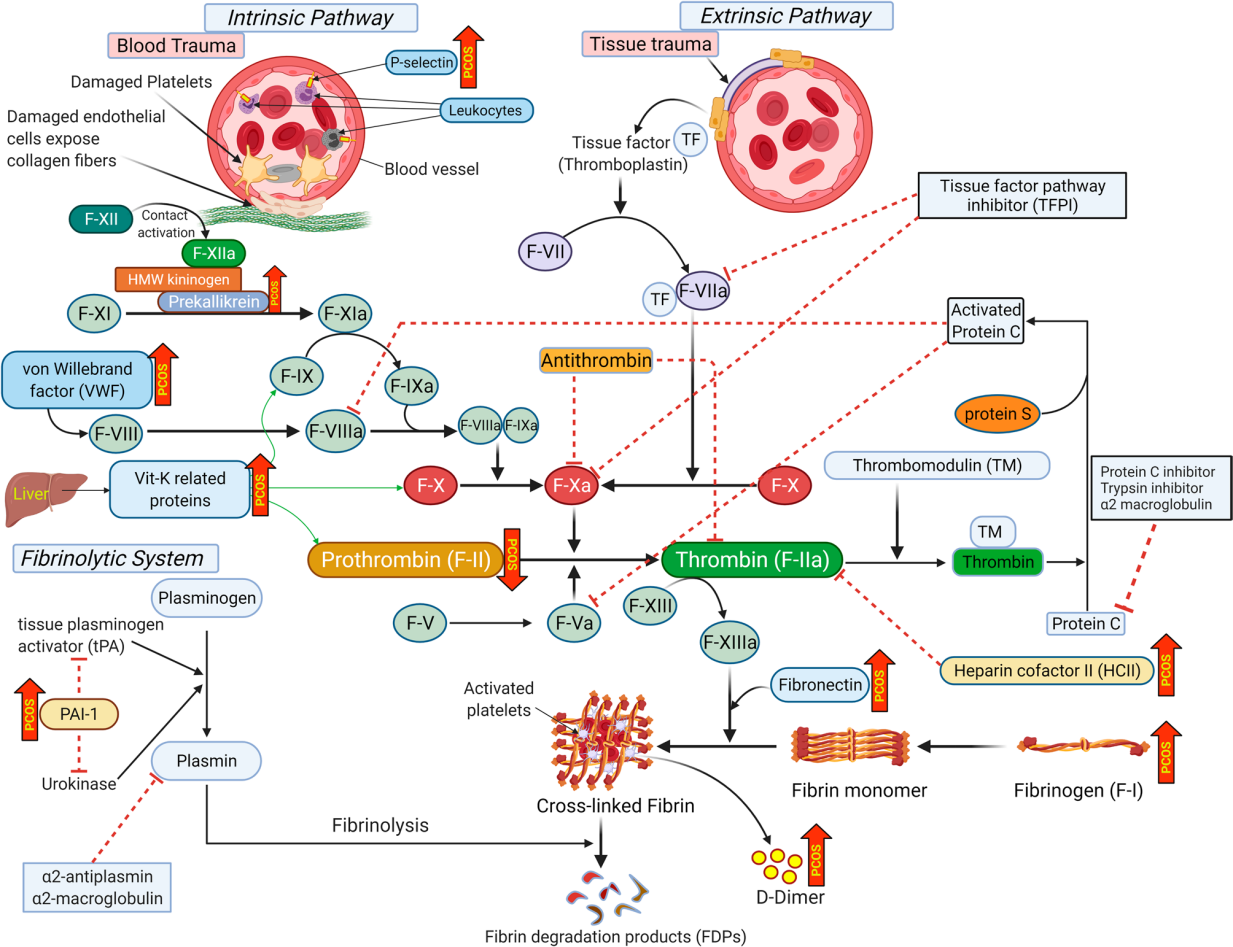
Schematic illustration showing altered coagulation pathway proteins in polycystic ovary syndrome (PCOS). Cellular stimulation factors and proteins involved in blood coagulation pathways [intrinsic (top left), extrinsic (top right) and fibrinolytic system (bottom left)] are illustrated. Proteins increased in women with PCOS are indicated by upward red arrows (labelled ‘PCOS’) with the protein decreased in PCOS (prothrombin) indicated by a downward red arrow. Red dotted lines indicate the inhibitory actions of the proteins in blood coagulation pathways.

Pro-coagulation proteins elevated in PCOS are shown in Fig. 1 and include plasminogen activator inhibitor-1 (PAI-1) (2259 ± 137 vs 1457 ± 107 RFU, PCOS vs control, *p* < 0.0001), fibrinogen (177,423 ± 2108 vs 169,230 ± 2425 RFU, PCOS vs control, *p* < 0.01), fibrinogen gamma chain (63,118 ± 946 vs 57,328 ± 830 RFU, *p* < 0.0001), fibronectin (24,594 ± 2627 vs 16,041 ± 698 RFU, *p* < 0.01), von Willebrand factor (19,849 ± 3038 vs 13,159 ± 595 RFU, *p* < 0.05), D-dimer (13,860 ± 185 vs 12,708 ± 172 RFU, *p* < 0.0001), P-selectin (13,843 ± 317 vs 12,660 ± 412 RFU, *p* < 0.05), and plasma kallikrein (24,868 ± 376 vs 22,981 ± 447 RFU, *p* < 0.001). Prothrombin levels were decreased in PCOS (161,458 ± 1275 vs 165,233 ± 1958 RFU, *p* < 0.05) and the anticoagulant vitamin K-dependent protein S (4403 ± 69 vs 3989 ± 59 RFU, *p* < 0.0001) and heparin cofactor II (HCII) (4156 ± 64 vs 3821 ± 63 RFU, *p* < 0.001) were increased (Fig. 2). A schematic illustration of all the blood coagulation proteins altered in PCOS is shown in Fig. 3.

Significant correlations of coagulation proteins with body mass index are shown in Table 2 and include antithrombin III (*p* < 0.0001), fibrinogen gamma chain (*p* < 0.0001), vitamin K dependent protein S (*p* < 0.001), D-dimer (*p* < 0.001), fibronectin (*p* < 0.001), prothrombin (*p* < 0.01), heparin cofactor 2 (*p* < 0.01), fibrinogen (*p* < 0.05), angiostatin (*p* < 0.05), fibrinogen fragment 3 (*p* < 0.05) and P-selectin (*p* < 0.05). Significant correlations of coagulation proteins with CRP, as a marker of inflammation, are shown in Table 2, that included antithrombin III (*p* < 0.0001), heparin cofactor 2 (*p* < 0.0001), fibrinogen gamma chain (*p* < 0.0001), D-dimer (*p* < 0.0001), P-selectin (*p* < 0.001), fibronectin (*p* < 0.001), and its fragments 3 and 4 (*p* < 0.01, respectively), vitamin K dependent protein S, alpha 2 antiplasmin and fibrinogen (*p* < 0.05, respectively). Significant correlations of coagulation proteins with insulin resistance, as determined by HOMA-IR, were also seen for antithrombin III (*p* < 0.0001), heparin cofactor 2 (*p* < 0.001), P-selectin (*p* < 0.0001), fibronectin (*p* < 0.01), vitamin K dependent protein S and alpha 2 antiplasmin (*p* < 0.05, respectively) (Table 2). However, in a multivariate analysis accounting for BMI, inflammation (CRP) and insulin resistance (HOMA-IR), there was no correlation with the coagulation proteins (Table 2).

To eliminate the confounding effect of obesity, a subset of women with BMI ≤ 25 kg/m^2^ (16 PCOS and 53 controls) were compared. Here, HOMA-IR remained elevated in PCOS (1.6 ± 1.2 vs 1.1 ± 0.5, *p* < 0.05), CRP did not differ, whilst heparin cofactor 2 (3979 ± 649 vs 3613 ± 585 RFU, *p* < 0.05) was elevated in the normal weight PCOS group (Table 3).

**Table 3:** Demographics, baseline, hormonal and metabolic parameters of the normal weight (BMI ≤ 25 kg/m^2^) PCOS subjects and controls (16 PCOS and 53 control women).

Baseline demographics	PCOS (n = 16)	Controls (n = 53)	*p* value
	Mean (SEM)	Mean (SEM)	
Age (years)	27.8 (1.7)	29.5 (0.9)	0.37
BMI (Kg/m^2^)	22.7 (0.5)	22.8 (0.3)	0.87
Weight (Kg)	64.1 (1.9)	63.0 (1.0)	0.59
Insulin (IU/ml)	8.9 (2.4)	5.0 (0.5)	0.02
HOMA-IR	1.6 (0.3)	1.1 (0.09)	0.04
CRP (mg/L)	1.2 (0.2)	0.9 (0.1)	0.22
SHBG (nmol/L)	72.7 (15.6)	95.9 (12.2)	0.33
Testosterone (nmol/l)	1.2 (0.1)	1.1 (0.1)	0.29

*BMI* Body mass index, *HOMA-IR* Homeostasis model of assessment-insulin resistance, *CRP* C reactive protein, *SHBG* Sex hormone binding globulin.

## Discussion

These data show that the hypercoagulable state in PCOS can be completely accounted for by BMI and its associated inflammation, and enhanced insulin resistance. In comparison to the normal controls, overall 10 pro-coagulation proteins were elevated in PCOS; plasminogen activator inhibitor-1 (PAI-1), fibrinogen, fibrinogen gamma chain, fibronectin, von Willebrand factor, D-dimer, P-selectin, plasma kallikrein. anticoagulant vitamin K-dependent protein S and heparin cofactor II, whilst prothrombin was decreased. These results are in accord with others who have reported changes in coagulation proteins in PCOS^4,13^, but underlying pathophysiology has not been previously described and shows that in PCOS alterations in the coagulation factors appears complex and multifactorial. When normal weight (BMI ≤ 25) PCOS patients were compared with normal weight control subjects, insulin resistance remained elevated and heparin cofactor 2, that is protective and inactivates thrombin in tissues, differed. These data are in accord with the association of heparin cofactor 2 with insulin resistance^14^, indicating that normal weight PCOS subjects likely have no additional risk associated with a hypercoagulable state; however, obesity with associated inflammation markedly exaggerates the hypercoagulable state with an increased number of clotting parameters altered. The multivariate analysis showed that all of the changes in the coagulation proteins could be accounted for by BMI, inflammation and insulin resistance.

It is well recognized that obesity causes inflammation and increased insulin resistance^15,16^ and is associated with changes in coagulation parameters. For example, fibronectin is correlated to BMI^17^ and obesity is associated with increased PAI-1 in PCOS^18^. Others have reported, using a repeated fibrin formation and degradation functional assay, that “overall hemostatic potential” was BMI-dependent and not associated with PCOS^19^. Central fat mass has been associated with fibrinogen, CRP, coagulation factor XIII, waist-to-hip ratio, plasminogen, PAI-1, plasmin inhibitor, and thrombin activatable fibrinolysis inhibitor^20^).

Conversely, thrombin-activatable fibrinolysis inhibitor, PAI-1, D-dimer, Antithrombin III and thrombomodulin were reported to be significantly increased in women with PCOS compared with age- and BMI-matched controls, suggesting that alterations in these proteins are BMI-independent and due to other factors such as inflammation and insulin resistance, as reported here^4^.

Inflammation (CRP) correlated significantly with antithrombin III, heparin cofactor 2, fibrinogen gamma chain, D-dimer, P-selectin, fibronectin, and its fragments 3 and 4, vitamin K dependent protein S, alpha 2 antiplasmin and fibrinogen. Inflammation crosstalk with coagulation leading to increased coagulopathy is well recognized; however, with the initiation of coagulation, the coagulation proteases may then modulate the inflammatory response^21,22^. In PCOS, both CRP and fibrinogen are predicted by BMI in accord with obesity initiating the increased inflammation^23^ and particularly CRP, PAI-1, D-dimer, Antithrombin III with central fat mass as noted above^20^.

In this study, insulin resistance (HOMA-IR) correlated with Antithrombin III, heparin cofactor 2, P-selectin, fibronectin, vitamin K dependent protein S and alpha 2 antiplasmin. It is recognized that insulin resistance is associated with enhanced thrombogenesis^24^; however, it is difficult to determine the contribution of insulin resistance alone to its association with obesity and inflammation metabolic syndrome lipid parameters^25–27^.

As noted above, there are reports of changes in coagulation proteins in PCOS^4,13^ and changes in functional assays^2,3^; however, conversely others have not found changes in the coagulation proteins between PCOS and controls^28^. It can be seen from the data presented here that the likely reason for these discrepancies are due to the patient population being studied with the results dependent on the degree of obesity, inflammation and insulin resistance present. In addition, the PCOS phenotype may have an important role, with those having all three of the diagnostic criteria exhibiting the metabolic phenotype with increased insulin resistance in comparison to those with only two of the three diagnostic criteria^29^.

The hypercoagulation state is in homeostasis with the pro-coagulation protein changes seen here in PCOS being balanced by the reduction in prothrombin and increased vitamin K-dependent protein S and heparin cofactor II that we also report.

Limitations of this study include that it was a cross sectional study, but this was mitigated by the large number of subjects. As all study subjects were Caucasian, these results may not be generalizable to other ethnic populations. Only BMI, rather than an assessment of visceral fat, was available for this study population. In addition, only the proteins involved in the coagulation cascade and fibrinolysis pathway were measured. It would have been optimal to have included prothrombin fragments 1 and 2 as markers of activated coagulation, but these were not available in the SOMAscan proteomics panel. Likewise, the thrombin generation assay would have been a good indicator of an increase in activated coagulation; however, no functional assays were undertaken in this study. Whilst there was no single good marker for activated coagulation it has well been recognised that indicators found here, such as high plasma fibrinogen, factor VII/VIIa, tissue-type plasminogen activator and plasminogen activator inhibitor levels, have been associated with at least as great a risk of developing myocardial (re)infarction or sudden death as high cholesterol levels, especially in the young^11^.

In conclusion, the hypercoagulable state in PCOS can be fully accounted for by BMI, inflammation and insulin resistance. This is an important finding because, whilst the prothrombotic propensity of women with PCOS has previously been reported, it was assumed to be intrinsic to the PCOS condition. Here, we show that this is, in fact, not the case, but is a consequence of the increased BMI, inflammation and insulin resistance that often accompany the PCOS condition; the impact of directed therapy needs to be determined in the future. Further studies in lean PCOS women would help to clarify our findings.

## Data Availability

All the data for this study will be made available upon reasonable request to the corresponding author.
